# Evaluation of the neonatal sequential organ failure assessment and mortality risk in neonates with respiratory distress syndrome: A retrospective cohort study

**DOI:** 10.3389/fped.2022.911444

**Published:** 2022-07-22

**Authors:** Shanshan Shi, Jie Guo, Minqiang Fu, Lihua Liao, Jiabin Tu, Jialing Xiong, Quanwang Liao, Weihua Chen, Kaihong Chen, Ying Liao

**Affiliations:** ^1^Longyan First Affiliated Hospital of Fujian Medical University, Longyan, China; ^2^The Third Clinical Medicine College, Fujian Medical University, Fuzhou, China

**Keywords:** neonatal sequential organ failure assessment, neonate, respiratory distress syndrome, critical care, mortality

## Abstract

**Background:**

Respiratory distress syndrome (RDS) is one of the leading causes of neonatal death in the neonatal intensive care unit (NICU). Previous studies have suggested that the development of neonatal RDS may be associated with inflammation and lead to organ dysfunction. The neonatal sequential organ failure assessment (nSOFA) scoring system is an operational definition of organ dysfunction, but whether it can be used to predict mortality in neonates RDS is unknown. The aim of this study was to clarify the performance of the nSOFA score in predicting mortality in patients with neonatal RDS, with the aim of broadening the clinical application of the nSOFA score.

**Methods:**

Neonates with RDS were identified from the Medical Information Mart for Intensive Care (MIMIC)-III database. Cox proportional hazards model were used to assess the association between nSOFA score and mortality. Propensity score matched analysis were used to assess the robustness of the analytical results.

**Results:**

In this study of 1,281 patients with RDS of which 57.2% were male, death occurred in 40 cases (3.1%). Patients with high nSOFA scores had a higher mortality rate of 10.7% compared with low nSOFA scores at 0.3%. After adjusting for confounding, multivariate Cox proportional risk analysis showed that an increase in nSOFA score was significantly associated with increased mortality in patients with RDS [adjusted Hazards Ratio (aHR): 1.48, 95% Confidence Interval (CI): 1.32–1.67; *p* < 0.001]. Similarly, the High nSOFA group was significantly associated with higher mortality in RDS patients (aHR: 19.35, 95% CI: 4.41–84.95; *p* < 0.001) compared with the low nSOFA group.

**Conclusion:**

The nSOFA score was positively associated with the risk of mortality in cases of neonatal RDS in the NICU, where its use may help clinicians to quickly and accurately identify high risk neonates and implement more aggressive intervention.

## Introduction

Respiratory distress syndrome (RDS) is a clinical syndrome characterized by respiratory distress and progressive aggravation in affected neonates soon after birth and is one of the most common clinical conditions dealt with in the neonatal intensive care unit (NICU) (1). It is estimated to affect up to 7% of neonates and is among the major causes of neonatal mortality (2–4).

Previous studies have suggested that the pathogenesis of neonatal RDS is more complex than commonly recognized and inflammation may be involved (5). Nupponen et al. demonstrated a systemic inflammatory reaction in preterm infants with RDS (6). In recent studies, infection and inflammation have been identified as risk factors for RDS, which can cause organ dysfunction (5, 7, 8).

The neonatal sequential organ failure assessment (nSOFA) scoring system is an operational definition of organ dysfunction, which is widely used to identify the presence of life-threatening organ dysfunction among preterm infants with inflammation-related diseases such as infection and sepsis (9, 10).

This study was developed to clarify whether nSOFA score could be used to predict mortality in patients with neonatal RDS. In order to broaden the clinical application of the nSOFA score and to find a new method to assist clinicians to quickly and accurately identify high-risk neonates that require more aggressive intervention.

## Methods

### Study design

This is a retrospective cohort study with data from the Marketplace for Medical Information in Intensive Care III (MIMIC-III) database, a longitudinal single-center database that contains information related to patients admitted to the intensive care unit at Beth Israel Deaconess Medical Center (Boston, MA, United States) between 2001 and 2012 (11). The database is maintained by the Massachusetts Institute of Technology (MIT) Computational Physiology Laboratory. The project was approved by the institutional review boards of MIT and the Beth Israel Deaconess Medical Center (BIDMC) and was granted a waiver of informed consent. After successfully completing the National Institutes of Health (NIH) web-based training course and the Protecting Human Research Participants examination (no. 41897755), permission was given to extract data from MIMIC-III.

### Selection of participants

The MIMIC-III database contains a total of 7,870 patients admitted to the NICU and patients who didn’t develop RDS were excluded. A total of 1,281 patients with RDS were included in the final study cohort, grouped according to nSOFA score level and survival status, respectively. The flow chart of patient screening is shown in Supplementary Figure 1. The diagnosis of RDS was carried out according to the Canadian neonatal network (CNN) (4) and defined as babies requiring respiratory support exceeding 24 h, intubation, surfactant administration (but not for meconium aspiration, pneumonia, or pulmonary hemorrhage), fraction of inspired oxygen (FiO_2_) exceeding 25% for a minimum of 24 h or according to the international statistical classification of diseases and related health problem ninth revision (ICD-9) codes including code 769. For patients who were admitted to the NICU more than once, only the first NICU stay was included for analysis.

### Variable extraction

Clinical data were extracted from the MIMIC-III database for the first 24 hours of the patient’s NICU admission, including demographics, vital signs, laboratory tests, diagnostic codes, medications and survival data. Clinical data required to calculate the nSOFA score were also collected and included the receipt of intubation and mechanical ventilation, the FiO_2_ to achieve the peripheral oxygen saturation (SpO_2_), the platelet count and any requirement for glucocorticoid, inotropic, or vasoactive drugs. The SpO_2_/FiO_2_ were converted using the partial pressure of arterial oxygen (PaO_2_)/FiO_2_ ratio conversion where SpO_2_/FiO_2_ = 64 + 0.84 × (PaO_2_/FiO_2_) (12). Missing values in the nSOFA score were assumed to be in the normal range as in previous studies (13, 14) and if a variable was recorded more than once in the first 24 h, the most serious record was used. Comparison of baseline characteristics of patients with missing and non-missing SpO_2_/FiO_2_ is shown in Supplementary Table 1. Comorbidities identified based on documenting the ICD-9 codes included hemolytic disease of the newborn (HDN), congenital heart disease (CHD), acidosis, anemia, bleed, pneumonia, jaundice, and septicemia. Acute kidney injury (AKI) was defined according to the Kidney Disease: Improving Global Outcomes (KDIGO) guidelines as an increase in serum creatinine (Scr) of 0.3 mg/dL or more from baseline within 48 h (15).

The primary outcome of this study was classified as all-cause mortality. Inpatient mortality information was obtained from the Hospital Information System, and mortality information for discharged patients was obtained from the US Social Security Death Index.

### Application of the neonatal sequential organ failure assessment score

The nSOFA score uses categorical scores with a total score range from 0 as best to 15 as worst to objectively describe (1) receipt of mechanical ventilation and oxygen to maintain a physiologic peripheral saturation which was scored 0 to 8; (2) inotropic or vasoactive drug support, including the use of corticosteroids for presumed adrenal insufficiency or catecholamine-resistant shock scored 0 to 4; and (3) the presence and severity of thrombocytopenia based on the most recent platelet measure scored 0 to 3 (9) (Table 1).

**TABLE 1:** Neonatal sequential organ failure assessment (nSOFA) components and scoring.^a^

Component	nSOFA scores				
**Respiratory score**	0	2	4	6	8
Criteria	Not intubated or intubated, SpO_2_/FiO_2_ ≥ 300	Intubated, SpO_2/_FiO_2_ < 300	Intubated, SpO_2_/FiO_2_ < 200	Intubated, SpO_2_/FiO_2_ < 150	Intubated, SpO_2_/FiO_2_ < 100
**Cardiovascular score**	0	1	2	3	4
Criteria^b^	No inotropes and no systemic corticosteroid treatment	No inotropes and systemic corticosteroid treatment	1 inotrope and no systemic corticosteroid treatment	≥ 2 inotropes or 1 inotrope and systemic corticosteroid treatment	≥2 inotropes and systemic corticosteroid treatment
**Hematologic score**	0	1	2	3	NA
Criteria^c^	Platelet count* ≥ 150 × 10^3^	Platelet count 100–149 × 10^3^	Platelet count < 100 × 10^3^	Platelet count < 50 × 10^3^	

nSOFA, feonatal sequential organ failure assessment; FiO_2_, fraction of inspiratory oxygen; SpO_2_, peripheral oximetric saturation.

*SI conversion factor: To convert platelet count to × 10^9^/L, multiply by 1.

^a^Score range, 0 (best) to 15 (worst).

^b^Medications considered as inotropic or vasoactive included dopamine, dobutamine, epinephrine, norepinephrine, vasopressin, and phenylephrine.

^c^Most recent platelet count available to the clinician.

### Statistical analysis

Values are presented as the means ± standard deviations or medians as interquartile ranges (IQRs) for continuous variables and categorical variables are presented as total numbers and percentages. Comparisons between groups were made using the Student’s *t-*test or the Mann-Whitney U test for continuous variables and χ^2^-test or Fisher’s exact test for categorical variables.

The area under the receiver operating characteristic curve (AUROC) was calculated to assess the accuracy of the nSOFA score and its subscales in predicting the primary outcome in patients with RDS. The cut-off values of the receiver operating characteristic (ROC) curve were identified by calculating the Youden index, which divided the high and low nSOFA groups. The Kaplan-Meier survival analysis were performed to evaluate the incidence rate of primary outcome events among groups according to various levels of the nSOFA score and discrepancies among groups were evaluated by log-rank test. Cox proportional hazards models were used to estimate the relationships between nSOFA score (per 1 score)/nSOFA groups (high nSOFA groups and low nSOFA groups) and primary outcomes. Characteristic variables with significant baseline differences or clinical significance were used as candidate predictors in the multivariate Cox regression model. Variables with more than 20% missing data were excluded from the analysis. The details of missing data are summarized in Supplementary Table 3.

The propensity score matching (PSM) was used to adjust for ethnicity, sex, and gestational age to ensure the comparability across of high and low nSOFA groups and between survival and non-survival groups in the analysis of baseline characteristics. Baseline characteristics of the original and matched cohorts were presented separately. Multivariate Cox regression analysis was also performed on the matching cohort. Subgroup analyses was used to investigate whether the risk was modified by sex and septicemia. All analyses were first unadjusted as model one, then adjusted for sex, ethnicity, gestational age, weight, and heart rate to get model two and finally for anemia, bleed, septicemia, and pulmonary surfactant to get model three.

All data analyses were performed using R software (version 4.0.4; R Foundation for Statistical Computing, Vienna, Austria) and SPSS statistical software (SPSS Statistics 24.0). Bilateral *p*-values < 0.05 for all analyses were considered statistically significant.

## Results

### Baseline characteristics

There were 1,281 patients with RDS who were analyzed between 2001 and 2012 of whom 733 (57.2%) were male and 1,127 (93.1%) were less than 37 weeks of gestational age. There were 40 (3.1%) patient deaths in this cohort. The optimal cut-off value for nSOFA score associated with death was defined as 2.5 and this was used to divide the cohort into low and high nSOFA groups.

There was a total of 347 (27.1%) patients with a high nSOFA score, of whom 37 (10.7%) died, compared with 3 (0.3%) of the 934 (72.9%) patients with a low nSOFA score. Patients in the high nSOFA group had more gestational age less than 28 weeks, smaller height, weight and head circumference, lower SpO_2_/FiO_2_, less urine output, additional complications such as acute kidney injury, bleeding and infection and had higher rates of ventilator and vasoactive drug use on the first day of admission (all *p* < 0.05). Baseline information for the original cohort and matched cohort of RDS patients with high and low nSOFA scores is shown in Table 2. Baseline characteristics of grouping according to the occurrence of follow-up deaths is shown in Supplementary Table 4.

**TABLE 2:** Demographic data and comparisons between the low and high nSOFA groups.

Characteristic	Original cohort			*P*-value	Matched cohort			*P*-value
	Overall	Low nSOFA	High nSOFA^a^		Overall	Low nSOFA	High nSOFA	
				
	(*N* = 1281)	(*N* = 934)	(*N* = 347)		(*N* = 626)	(*N* = 313)	(*N* = 313)	
**Demographic**								
Ethnicity, n (%)				0.334				0.987
Asian	48 (3.7)	38 (4.1)	10 (2.9)		17 (2.7)	9 (2.9)	8 (2.6)	
Black	148 (11.6)	105 (11.2)	43 (12.4)		66 (10.5)	32 (10.2)	34 (10.9)	
Hispanic/Latino	55 (4.3)	41 (4.4)	14 (4.0)		24 (3.8)	11 (3.5)	13 (4.2)	
White	849 (66.3)	628 (67.2)	221 (63.7)		428 (68.4)	216 (69.0)	212 (67.7)	
Other	181 (14.1)	122 (13.1)	59 (17.0)		91 (14.5)	45 (14.4)	46 (14.7)	
Sex, male, n (%)	733 (57.2)	529 (56.6)	204 (58.8)	0.530	376 (60.1)	189 (60.4)	187 (59.7)	0.935
Gestational age, n (%)				<0.001				1.000
23–25 weeks	35 (2.9)	5 (0.6)	30 (9.1)		10 (1.7)	5 (1.7)	5 (1.7)	
26–28 weeks	101 (8.3)	48 (5.4)	53 (16.2)		90 (15.3)	45 (15.3)	45 (15.3)	
29–31 weeks	346 (28.6)	246 (27.9)	100 (30.5)		200 (34.0)	100 (33.9)	100 (34.0)	
32–35 weeks	642 (53.1)	526 (59.6)	116 (35.4)		232 (39.4)	116 (39.3)	116 (39.5)	
36 weeks	3 (0.2)	3 (0.3)	0 (0.0)		0 (0.0)	0 (0.0)	0 (0.0)	
37 weeks	69 (5.7)	46 (5.2)	23 (7.0)		47 (8.0)	24 (8.1)	23 (7.8)	
38 weeks	1 (0.1)	1 (0.1)	0 (0.0)		0 (0.0)	0 (0.0)	0 (0.0)	
39 weeks	0 (0.0)	0 (0.0)	0 (0.0)		0 (0.0)	0 (0.0)	0 (0.0)	
≥ 40 weeks	13 (1.1)	7 (0.8)	6 (1.8)		10 (1.7)	5 (1.7)	5 (1.7)	
Height, cm	40.70 (6.35)	41.60 (5.68)	38.33 (7.36)	<0.001	39.80 (6.47)	40.57 (5.41)	39.05 (7.29)	0.004
Weight, kg	1.64 (0.74)	1.75 (0.71)	1.37 (0.76)	<0.001	1.53 (0.76)	1.64 (0.76)	1.44 (0.75)	0.001
Head circumference, cm	28.73 (3.72)	29.41 (3.23)	26.93 (4.28)	<0.001	28.05 (3.84)	28.71 (3.36)	27.41 (4.15)	<0.001
**Vital signs**								
PaO_2_/FiO_2_	242.68 (146.09)	284.07 (143.45)	177.40 (125.00)	<0.001	216.93 (138.17)	280.66 (129.12)	178.11 (128.96)	<0.001
SpO_2_/FiO_2_	267.85 (122.71)	302.62 (120.50)	213.02 (105.00)	<0.001	246.22 (116.06)	299.75 (108.46)	213.61 (108.33)	<0.001
HR, bmp	145.72 (10.04)	144.32 (9.11)	149.35 (11.36)	<0.001	146.70 (10.50)	144.63 (9.38)	148.68 (11.14)	<0.001
Respirate,/minute	44.93 (16.21)	45.89 (16.08)	42.38 (16.28)	0.001	43.93 (16.98)	45.47 (17.29)	42.44 (16.56)	0.028
Urine output, mL/d	87.41 (45.54)	91.86 (44.78)	75.83 (45.53)	<0.001	82.13 (44.46)	84.76 (43.19)	79.61 (45.57)	0.165
**Laboratory tests**								
RBC, m/μL	4.23 (0.67)	4.30 (0.65)	4.03 (0.69)	<0.001	4.16 (0.65)	4.26 (0.58)	4.05 (0.71)	<0.001
Hemoglobin, g/dL	15.73 (2.23)	15.90 (2.11)	15.26 (2.49)	<0.001	15.59 (2.32)	15.86 (2.05)	15.32 (2.54)	0.004
RDW,%	17.09 (1.36)	17.10 (1.19)	17.08 (1.75)	0.858	17.13 (1.55)	17.08 (1.25)	17.17 (1.80)	0.468
Hematocrit,%	46.49 (7.07)	47.31 (6.49)	44.31 (8.04)	<0.001	45.88 (7.50)	47.17 (6.52)	44.58 (8.19)	<0.001
WBC, K/μL	10.82 (6.21)	11.20 (6.01)	9.82 (6.62)	<0.001	10.61 (6.93)	11.27 (7.04)	9.94 (6.78)	0.017
Lymphocyte,%	58.02 (17.86)	57.78 (17.92)	58.64 (17.69)	0.450	57.44 (18.31)	55.84 (18.86)	59.05 (17.63)	0.029
Neutrophil,%	29.60 (16.32)	30.27 (16.51)	27.82 (15.67)	0.018	29.64 (16.43)	31.72 (16.94)	27.56 (15.65)	0.002
Platelet, K/uL	256.37 (83.37)	269.00 (77.61)	222.51 (88.78)	<0.001	242.88 (87.81)	264.82 (80.37)	220.73 (89.54)	<0.001
Bilirubin, mg/dL	5.12 (1.92)	5.38 (1.82)	4.40 (2.01)	<0.001	4.92 (1.94)	5.22 (1.85)	4.62 (1.99)	<0.001
Serum potassium, mEq/L	4.97 (1.07)	5.04 (1.09)	4.78 (0.98)	<0.001	4.92 (1.04)	5.06 (1.07)	4.78 (1.00)	0.001
Serum sodium, mEq/L	138.76 (4.99)	138.44 (4.81)	139.65 (5.37)	<0.001	138.91 (4.80)	138.54 (4.46)	139.28 (5.10)	0.063
**Comorbidities**								
AKI (48h), n (%)	52 (4.1)	34 (3.6)	18 (5.2)	0.277	29 (4.6)	13 (4.2)	16 (5.1)	0.704
HDN, n (%)	14 (1.1)	10 (1.1)	4 (1.2)	1.000	6 (1.0)	3 (1.0)	3 (1.0)	1.000
CHD, n (%)	52 (4.1)	40 (4.3)	12 (3.5)	0.613	23 (3.7)	13 (4.2)	10 (3.2)	0.671
Acidosis, n (%)	84 (6.6)	40 (4.3)	44 (12.7)	<0.001	61(9.7)	21 (6.7)	40 (12.8)	0.015
Anemia, n (%)	294 (23.0)	191 (20.4)	103 (29.7)	0.001	156 (24.9)	70 (22.4)	86 (27.5)	0.166
Bleed, n (%)	33 (2.6)	17 (1.8)	16 (4.6)	0.009	18 (2.9)	8 (2.6)	10 (3.2)	0.811
Pneumonia, n (%)	26 (2.0)	14 (1.5)	12 (3.5)	0.047	16 (2.6)	5 (1.6)	11 (3.5)	0.205
Jaundice, n (%)	1105 (86.3)	811 (86.8)	294 (84.7)	0.378	537 (85.8)	273 (87.2)	264 (84.3)	0.360
Septicemia, n (%)	159 (12.4)	75 (8.0)	84 (24.2)	<0.001	107 (17.1)	37 (11.8)	70 (22.4)	0.001
**Medications**								
Ventilation time, hours	267.51 (458.79)	167.73 (323.45)	511.19 (621.42)	<0.001	376.19 (543.33)	251.43 (436.73)	489.75 (603.39)	<0.001
Mechanical ventilation, n (%)	971 (79.3)	661 (75.3)	310 (89.6)	<0.001	506 (83.2)	230 (77.7)	276 (88.5)	0.001
Methasone, n (%)	61 (4.8)	20 (2.1)	41 (11.8)	<0.001	43(6.9)	9 (2.9)	34 (10.9)	<0.001
Microbiotic, n (%)	831 (64.9)	593 (63.5)	238 (68.6)	0.103	419 (66.9)	209 (66.8)	210 (67.1)	1.000
Furosemide, n (%)	139 (10.9)	67 (7.2)	72 (20.7)	<0.001	90(14.4)	27 (8.6)	63 (20.1)	<0.001
Dopamine, n (%)	170 (13.3)	0 (0.0)	170 (49.0)	<0.001	145 (23.2)	0 (0.0)	145 (46.3)	<0.001
Dobutamine, n (%)	9 (0.7)	0 (0.0)	9 (2.6)	<0.001	7 (1.1)	0 (0.0)	7 (2.2)	0.023
Epinephrine, n (%)	2 (0.2)	0 (0.0)	2 (0.6)	0.127	2 (0.3)	0 (0.0)	2 (0.6)	0.479
Pulmonary surfactant, n (%)	143 (11.2)	99 (10.6)	44 (12.7)	0.342	72 (11.5)	38 (12.1)	34 (10.9)	0.707
**Events**								
Death, n (%)	40 (3.1)	3 (0.3)	37 (10.7)	<0.001	25(4.0)	2 (0.6)	23 (7.3)	<0.001
**Score**								
nSOFA	1.72 (2.33)	0.52 (0.85)	4.95 (1.92)	<0.001	2.71 (2.60)	0.55 (0.86)	4.86 (1.87)	<0.001
Respiratory score	1.16 (1.77)	0.45 (0.84)	3.07 (2.17)	<0.001	1.75 (2.10)	0.45 (0.84)	3.05 (2.17)	<0.001
Cardiovascular score	0.45 (1.07)	0.02 (0.14)	1.59 (1.54)	<0.001	0.76 (1.31)	0.03 (0.17)	1.50 (1.53)	<0.001
Hematological score	0.12 (0.44)	0.05 (0.24)	0.30 (0.71)	<0.001	0.19 (0.57)	0.07 (0.29)	0.31 (0.73)	<0.001

nSOFA, neonatal sequential organ failure assessment; FiO_2_, fraction of inspiratory oxygen; PaO_2_, partial pressure of arterial oxygen; SpO_2_, peripheral oxygen saturation; HR, heart rate; bmp, beats per minute; RBC, red blood cell; RDW, red cell distribution width; WBC, white blood cell; AKI, acute renal injury; HDN, hemolytic disease of the newborn; CHD, congenital heart disease.

^a^Low nSOFA group (≤ 2.5); High nSOFA group (> 2.5).

### Comparisons of neonatal sequential organ failure assessment score

**FIGURE 1:**
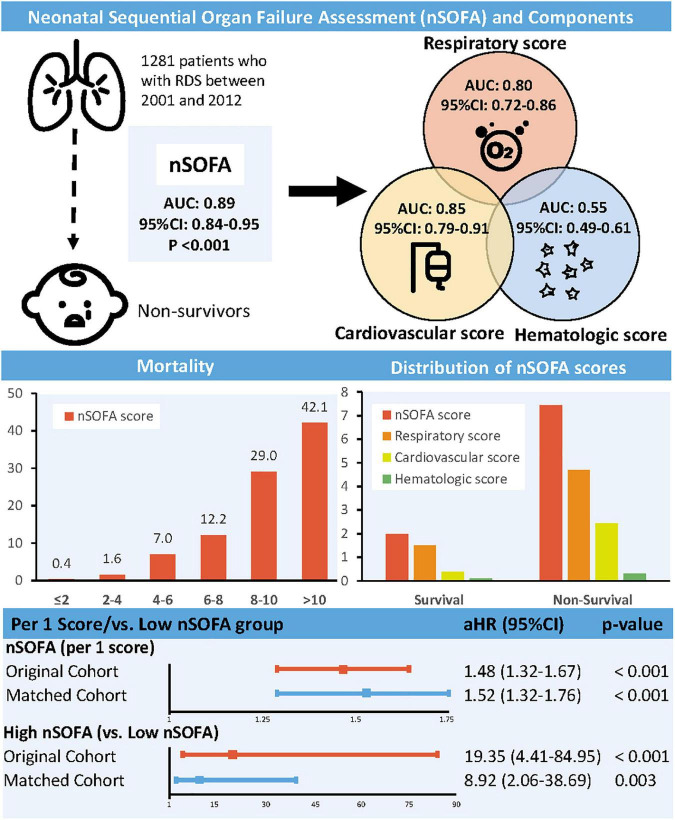
Association between neonatal sequential organ failure assessment and mortality. nSOFA, neonatal sequential organ failure assessment; RDS, respiratory distress syndrome; AUC, area under curve; CI, confidence interval; HR, hazard ratio.

Compared with survivors, non-survivors had higher nSOFA scores (6.20 vs. 1.58, *p* < 0.001) and subscales (respiratory score: 3.45 vs. 1.09; cardiovascular score: 2.45 vs. 0.38; hematologic score: 0.30 vs. 0.11). The mortality rate of RDS patients increased with increasing nSOFA score (0–2: 0.4%; 2–4: 1.6%; 4–6: 7.0%; 6–8: 12.2%; 8–10: 29.0%; 10–15: 42.1%). The area under curve (AUC) for the nSOFA score predicting death in patients with RDS was 0.89 (95% CI, 0.84–0.95, *p* < 0.001), which was higher than the predictive efficacy of the subscale [respiratory score: 0.80 (95% CI, 0.72–0.86, *p* < 0.001); cardiovascular score: 0.85 (95% CI, 0.79–0.91, *p* < 0.001); hematologic score: 0.55 (95% CI, 0.49–0.61, *p* = 0.690)] (Figure 1). The optimal cut-off value of the nSOFA score for predicting death in patients with RDS was 2.5, a sensitivity of 92.5% and a specificity of 75.0% (Supplementary Figure 2). We added gestational age to the original nSOFA score for scoring and analysis. The results showed that the AUC of the modified nSOFA score was 0.91 (95% CI, 0.86–0.95, *p* < 0.001), which was higher than that of the original nSOFA score and gestational age alone [0.73 (95% CI, 0.62–0.84, *p* < 0.001)].

### Association between neonatal sequential organ failure assessment scores and mortality

**FIGURE 2:**
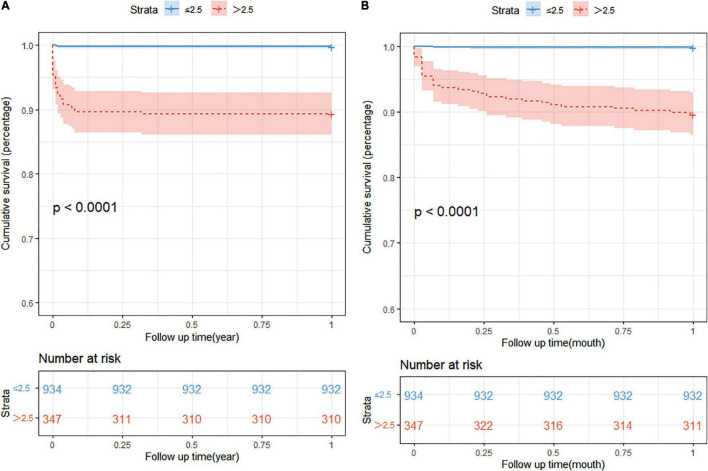
The Kaplan-Meier survival analysis curves. **(A)** The 1-year all-cause mortality; **(B)** 1-month all-cause mortality. nSOFA, neonatal sequential organ failure assessment.

The prognostic impact of nSOFA scores on patients who with RDS was studied. The Kaplan-Meier survival analysis curves for assessing the mortality between groups based on low and high nSOFA groups are shown in Figure 2. There was a statistically significant difference in mortality between low nSOFA and high nSOFA groups (log-rank *p* < 0.001). Univariate Cox proportional risk analysis showed that each unit increase in nSOFA scores was significantly associated with mortality (HR: 1.59, 95% CI: 1.46–1.73; *p* < 0.001). Even after adjusting for sex, ethnicity, gestational age, weight, heart rate, anemia, bleed, septicemia, and pulmonary surfactant, each unit score increase in nSOFA score remained significantly associated with mortality in patients with RDS (aHR: 1.48, 95% CI: 1.32–1.67; *p* < 0.001). Similarly, the high nSOFA group was significantly associated with higher mortality in RDS patients compared with the low nSOFA group (aHR: 19.35, 95% CI: 4.41–84.95; *p* < 0.001; Table 3).

**TABLE 3:** Association between nSOFA scores and mortality.

Categories	Event	Model 1		Model 2		Model 3	
		HR (95% CI)	*P*-value	HR (95% CI)	*P-value*	HR (95% CI)	*P*-value
**Original cohort**							
nSOFA (per 1 score)	40/1,281	1.59 (1.46–1.73)	<0.001	1.44 (1.29–1.61)	<0.001	1.48 (1.32–1.67)	<0.001
High (vs. Low) nSOFA **^a^**	37/347	52.56 (12.67–218.08)	<0.001	18.39 (4.21–80.35)	<0.001	19.35 (4.41–84.95)	<0.001
**Matched cohort**							
nSOFA (per 1 score)	25/626	1.52 (1.35–1.70)	<0.001	1.49 (1.30–1.71)	<0.001	1.52 (1.32–1.76)	<0.001
High (vs. Low) nSOFA	23/313	11.92 (2.81–50.54)	<0.001	9.17 (2.13–39.54)	0.003	8.92 (2.06–38.69)	0.003

nSOFA, neonatal sequential organ failure assessment; CI, confidence interval; HR, hazard ratio.

^a^Low nSOFA group (≤ 2.5); High nSOFA group (> 2.5).

Model 1, unadjusted.

Model 2, adjusted for sex, ethnicity, gestational age, weight, and heart rate.

Model 3, adjusted for anemia, bleed, septicemia, and pulmonary surfactant.

### Sensitivity analysis

Stratified and interaction analyses were used to evaluate whether the association differed by sex and septicemia. The results showed that the association between nSOFA score and mortality in RDS patients did not vary with sex and septicemia status (Supplementary Table 5). PSM did not change any of the conclusions (Table 3).

## Discussion

This retrospective study investigated the relationship between nSOFA score and the risk of death in neonates with RDS and found that the nSOFA score was independently associated with the risk of death in neonates with RDS. Each unit increase in nSOFA score correlated with a 48% increase in the risk of death in patients with RDS. The risk of death was higher in neonates in the high nSOFA group compared to the low nSOFA group and the relationship persisted after multivariable adjustment for traditional neonatal mortality risk factors.

The clinical condition RDS is one of the most common to be managed in the NICU. The incidence of RDS is inversely proportional to the gestational age of the neonate, with smaller and earlier neonates having more severe disease and it is the leading cause of morbidity and mortality in preterm neonates (16). Despite rapid advances in modern medicine, neonates who have developed RDS still have a high mortality rate (17–19) and there is an urgent need for a method to alert clinicians to intervene in such high risk neonates. However, there is still a lack of validated tools to predict the prognosis of neonates with RDS. Recent studies have shown that not just premature birth, multiple pregnancies, and low birth weight will increase the risk of neonatal RDS (5, 6). Infection and inflammation has been identified as a risk factor for RDS (5) and Nupponen et al. showed that neutrophils were activated in neonates with RDS (6). The nSOFA score is an operational definition of organ dysfunction that can identify those preterm neonates with infection and sepsis and an increased risk of mortality (9, 10), as sepsis is associated with a systemic inflammatory response which is implicated in the occurrence and development of RDS (6, 20). This study showed that the nSOFA score was strongly predictive of the mortality in neonates with RDS. As the first study to link nSOFA score with neonatal RDS, these results showed that the nSOFA score had a good predictive power for mortality risk in neonates with RDS and should be used for the assessment of prognostic risk in neonates with RDS.

In adults, the SOFA score includes aspects of central nervous system (CNS), liver dysfunction, and renal dysfunction, but these systems are more difficult to measure in neonates (10). The nSOFA score includes only three subscales defined as the respiratory, cardiovascular and hematological systems. The SpO_2_/FiO_2_ in the respiratory score is based on the Berlin definition to assess the severity of RDS, while mechanical ventilation maintains adequate ventilation and oxygenation to support organ function, both of which accurately reflect respiratory function. Corticosteroids have a wide range of potential effects in terms of anti-inflammatory, antioxidant, pulmonary vasodilatory and anti-edema actions (21), whereas vasoactive drugs optimize the hemodynamic status and improve blood perfusion to the organs (22). In the cardiovascular system, the use of vasoactive drugs and corticosteroids can be effective in assessing the severity of the disease. This study also observed that the cardiovascular subscale of the nSOFA score was the strongest predictor of neonatal death in RDS. According to a related article, coagulation dysfunction and progression of organ dysfunction in patients with thrombocytopenia have a significant correlation with each other (23), so the coagulation score based on platelet count can effectively reflect the progression of organ dysfunction. This study found that the hematological subscale of the nSOFA score appeared to perform poorly in predicting the prognosis of RDS neonates, whereas the nSOFA score demonstrated good predictive efficacy in terms of prognosis of RDS neonates. In addition, gestational age is known to be an independent predictor of incidence of RDS in neonates and is strongly associated with poor prognosis. Therefore, we added gestational age to the original nSOFA score for scoring and analysis. The results showed that the AUC of the modified nSOFA score was higher than that of the original score. This result provides a theoretical basis for the application of the nSOFA score in patients with RDS.

This study filled a gap in this field and broadens the clinical application of the nSOFA score, by determining the predictive power of the nSOFA score for mortality in RDS patients and its use to identify RDS patients at increased risk of adverse outcomes. As an objective measure of clinical course, nSOFA score has a potential utility both as a proxy variable for disease severity and as a risk assessment indicator for short- and long-term clinical endpoints (9). In this study, the nSOFA score showed good correlation in most cases and its findings will be useful for clinicians and researchers who need tools to objectively measure the risk of death, as well as for healthcare leaders and policy makers who develop resource allocation protocols for critically ill patients (24).

This study had several limitations. The data were only extracted from MIMIC-III data, which was a single-center retrospective study and it was difficult to control for a variety of confounders and bias in the analyses. In addition, individual patient-level data on treatment variables such as fluids, time to pulmonary surfactant and time to vasopressors was not available. Maternal medication data during pregnancy and delivery were not available. The SpO_2_/FiO_2_ was converted and replaced by using PaO_2_/FiO_2_. All the above factors may have influenced us to draw more definitive conclusions. Finally, this study did not examine the non-ICU population, so its applicability to neonates with suspected RDS in the rest of the hospital on the wards or in the Emergency Department could not be determined by this study. In the future, more large-scale, multicenter studies on neonatal mortality should be conducted to further verify these results.

## Conclusion

This study indicated that nSOFA score was associated with the risk of mortality in the neonatal RDS of NICU. The active use of the nSOFA score may help clinicians to quickly and accurately identify high risk neonates and implement more aggressive interventions.

## Data Availability Statement

The original contributions presented in the study are included in the article/Supplementary Material, further inquiries can be directed to the corresponding author/s.

## Ethics statement

The studies involving human participants were reviewed and approved by the Massachusetts Institute of Technology (Cambridge, MA) and Beth Israel Deaconess Medical Center (Boston, MA). Written informed consent to participate in this study was provided by the participants’ legal guardian/next of kin.

## Author contributions

YL conceptualized the research aims, planned the analyses, and guided the literature review. SS extracted the data from the MIMIC-III database. SS and JG participated in processing the data and performing the statistical analysis. LL, JT, JX, and WC wrote the first draft of the manuscript. SS, JG, MF, QL, KC, and YL provided comments and approved the final manuscript. All authors contributed to the article and approved the submitted version.

## Conflict of Interest

The authors declare that the research was conducted in the absence of any commercial or financial relationships that could be construed as a potential conflict of interest.

## Publisher’s Note

All claims expressed in this article are solely those of the authors and do not necessarily represent those of their affiliated organizations, or those of the publisher, the editors and the reviewers. Any product that may be evaluated in this article, or claim that may be made by its manufacturer, is not guaranteed or endorsed by the publisher.
