# Supplementary material for: Evaluation of the neonatal sequential organ failure assessment and mortality risk in neonates with respiratory distress syndrome: A retrospective cohort study
Source: Front Pediatr. 2022 Jul 22;10:911444. doi: 10.3389/fped.2022.911444 (PMC9352873; doi:10.3389/fped.2022.911444)
Supplement: Supplementary file 1 [file Data_Sheet_1.DOCX]

**Evaluation of the Neonatal Sequential Organ Failure Assessment and Mortality Risk in** **Neonates with Respiratory Distress Syndrome: A Retrospective Cohort Study**

Shanshan Shi, MD ^1,2†^, Jie Guo, MD ^1†^, Minqiang Fu, MD ^1†*^, Lihua Liao, MD ^1,2†^, Jiabin Tu, MD ^1,2^, Jialing Xiong, MD ^1,2^, Quanwang Liao, MD ^1^, Weihua Chen, MD ^1,2^, Kaihong Chen, MD ^1^, Ying Liao, MD ^1*^

^1^ Longyan First Affiliated Hospital of Fujian Medical University, Longyan, 364000, China

^2^ The Third Clinical Medicine College, Fujian Medical University, Fuzhou, 350000, China

**†These authors have contributed equally to this work and share first authorship**

*** Correspondence:**

Ying Liao, MD

Department of Cardiology, Longyan First Affiliated Hospital of Fujian Medical University, Longyan, 364000, China

Tel: (+86)13599638238/Fax: (+86)05972292374

E-mail: [wingjays@163.com](mailto:wingjays@163.com)

Minqiang Fu, MD

Department of Cardiology, Longyan First Affiliated Hospital of Fujian Medical University, Longyan, 364000, China

Tel: (+86)13605919623/Fax: (+86)05972292374

E-mail: [466045268@qq.com](mailto:466045268@qq.com)

**Table S1 Demographic Data and Comparisons between missing and non-missing SpO_2_/FiO_2_**

|  | **Overall** | **SpO2/FiO2** | **Non-SpO2/FiO2** |  |
| --- | --- | --- | --- | --- |
| **Characteristic** | (N=1,281) | (N=866) | (N=415) | ***P*-value** |
| **Demographic** |  |  |  |  |
| Ethnicity, n (%) |  |  |  | 0.226 |
| Asian | 48 (3.7) | 30 (3.5) | 18 (4.3) |  |
| Black | 148 (11.6) | 108 (12.5) | 40 (9.6) |  |
| Hispanic/Latino | 55 (4.3) | 38 (4.4) | 17 (4.1) |  |
| White | 849 (66.3) | 559 (64.5) | 290 (69.9) |  |
| Other | 181 (14.1) | 131 (15.1) | 50 (12.0) |  |
| Sex, male, n (%) | 733 (57.2) | 511 (59.0) | 222 (53.5) | 0.071 |
| Gestational age, n (%) |  |  |  | <0.001 |
| 23-25 weeks | 35 (2.7) | 32 (3.7) | 3 (0.7) |  |
| 26-28 weeks | 101 (7.9) | 94 (10.9) | 7 (1.7) |  |
| 29-31 weeks | 346 (27.0) | 257 (29.7) | 89 (21.4) |  |
| 32-35 weeks | 642 (50.1) | 379 (43.8) | 263 (63.4) |  |
| 36 weeks | 3 (0.2) | 3 (0.3) | 0 (0.0) |  |
| 37 weeks | 69 (5.4) | 52 (6.0) | 17 (4.1) |  |
| 38 weeks | 1 (0.1) | 0 (0.0) | 1 (0.2) |  |
| 39 weeks | 0 (0.0) | 0 (0.0) | 0 (0.0) |  |
| ≥ 40 weeks | 13 (1.0) | 7 (0.8) | 6 (1.4) |  |
| Height, cm | 40.70 (6.35) | 39.88 (6.80) | 42.46 (4.84) | <0.001 |
| Weight, kg | 1.64 (0.74) | 1.53 (0.74) | 1.89 (0.69) | <0.001 |
| Head circumference, cm | 28.73 (3.72) | 28.05 (3.87) | 30.19 (2.89) | <0.001 |
| **Vital Signs** |  |  |  |  |
| HR, bmp | 145.72 (10.04) | 146.27 (10.44) | 144.53 (9.02) | 0.005 |
| Respirate, /minute | 44.93 (16.21) | 43.44 (16.18) | 48.05 (15.83) | <0.001 |
| Urine output, ml/d | 87.41 (45.54) | 84.34 (44.92) | 94.32 (46.22) | 0.001 |
| **Laboratory Tests** |  |  |  |  |
| RBC, m/uL | 4.23 (0.67) | 4.17 (0.71) | 4.34 (0.57) | <0.001 |
| Hemoglobin, g/dL | 15.73 (2.23) | 15.59 (2.28) | 16.03 (2.10) | 0.001 |
| RDW, % | 17.09 (1.36) | 17.10 (1.47) | 17.08 (1.09) | 0.872 |
| Hematocrit, % | 46.49 (7.07) | 45.87 (7.27) | 47.82 (6.44) | <0.001 |
| WBC, K/uL | 10.82 (6.21) | 10.29 (6.42) | 11.95 (5.58) | <0.001 |
| Lymphocyte, % | 58.02 (17.86) | 57.80 (17.89) | 58.48 (17.80) | 0.526 |
| Neutrophil, % | 29.60 (16.32) | 29.70 (16.21) | 29.40 (16.57) | 0.763 |
| Platelet, K/uL | 256.37 (83.37) | 248.56 (82.37) | 272.85 (83.17) | <0.001 |
| Bilirubin, mg/dL | 5.12 (1.92) | 4.84 (1.90) | 5.70 (1.83) | <0.001 |
| Serum potassium, mEq/L | 4.97 (1.07) | 4.90 (1.06) | 5.13 (1.08) | 0.001 |
| Serum sodium, mEq/L | 138.76 (4.99) | 138.98 (5.25) | 138.29 (4.34) | 0.025 |
| **Comorbidities** |  |  |  |  |
| AKI (48h), n (%) | 52 (4.1) | 39 (4.5) | 13 (3.1) | 0.311 |
| HDN, n (%) | 14 (1.1) | 8 (0.9) | 6 (1.4) | 0.580 |
| CHD, n (%) | 52 (4.1) | 38 (4.4) | 14 (3.4) | 0.478 |
| Acidosis, n (%) | 84 (6.6) | 72 (8.3) | 12 (2.9) | <0.001 |
| Anemia, n (%) | 294 (23.0) | 236 (27.3) | 58 (14.0) | <0.001 |
| Bleed, n (%) | 33 (2.6) | 27 (3.1) | 6 (1.4) | 0.114 |
| Pneumonia, n (%) | 26 (2.0) | 22 (2.5) | 4 (1.0) | 0.097 |
| Jaundice, n (%) | 1105 (86.3) | 752 (86.8) | 353 (85.1) | 0.437 |
| Septicemia, n (%) | 159 (12.4) | 136 (15.7) | 23 (5.5) | <0.001 |
| **Medications** |  |  |  |  |
| Ventilation time, hours | 267.51 (458.79) | 335.77 (512.19) | 104.15 (221.05) | <0.001 |
| Mechanical ventilation, n (%) | 971 (79.3) | 673 (79.2) | 298 (79.7) | 0.902 |
| Methasone, n (%) | 61 (4.8) | 58 (6.7) | 3 (0.7) | <0.001 |
| Microbiotic, n (%) | 27 (2.1) | 27 (3.1) | 0 (0.0) | 0.001 |
| Furosemide, n (%) | 139 (10.9) | 121 (14.0) | 18 (4.3) | <0.001 |
| Dopamine, n (%) | 170 (13.3) | 163 (18.8) | 7 (1.7) | <0.001 |
| Dobutamine, n (%) | 9 (0.7) | 9 (1.0) | 0 (0.0) | 0.084 |
| Epinephrine, n (%) | 2 (0.2) | 2 (0.2) | 0 (0.0) | 0.823 |
| pulmonary surfactant, n (%) | 143 (11.2) | 118 (13.6) | 25 (6.0) | <0.001 |
| **Score** |  |  |  |  |
| nSOFA | 1.72 (2.33) | 2.48 (2.47) | 0.13 (0.55) | <0.001 |
| Respiratory score | 1.16 (1.77) | 1.72 (1.92) | 0.00 (0.00) | <0.001 |
| Cardiovascular score | 0.45 (1.07) | 0.63 (1.23) | 0.06 (0.40) | <0.001 |
| Hematological score | 0.12 (0.44) | 0.13 (0.47) | 0.08 (0.37) | 0.030 |
| **Events** |  |  |  |  |
| Death, n (%) | 40 (3.1) | 39 (4.5) | 1 (0.2) | <0.001 |

**Abbreviations:** FiO_2_: fraction of inspiratory oxygen; PaO_2_: partial pressure of arterial oxygen; SpO_2_: peripheral oxygen saturation; HR, heart rate; bmp, beats per minute; RBC, red blood cell; RDW, red cell distribution width; WBC, white blood cell; GLU, glucose; TG, triglyceride; ALP, alkaline phosphatase; BUN, blood urea nitrogen; INR, International Normalized Ratio; PT, prothrombin time; PH, pondus hydrogenii; PaCO_2_, partial pressure of carbon dioxide; AKI, acute renal injury; HDN, hemolytic disease of the newborn; CHD, congenital heart disease; nSOFA, neonatal sequential organ failure assessment.

**Table S2 Modified Neonatal Sequential Organ Failure Assessment (m-nSOFA) Components and Scoring.^a^**

| **Component** | **nSOFA Scores** | | | | |
| --- | --- | --- | --- | --- | --- |
| **Respiratory score** | 0 | 2 | 4 | 6 | 8 |
| Criteria | Not intubated or intubated, SpO_2_/FiO_2_≥300 | Intubated, SpO_2/_FiO_2_<300 | Intubated, SpO_2_/FiO_2_<200 | Intubated, SpO_2_/FiO_2_<150 | Intubated, SpO_2_/FiO_2_<100 |
| **Cardiovascular score** | 0 | 1 | 2 | 3 | 4 |
| Criteria^b^ | No inotropes and no systemic corticosteroid treatment | No inotropes and systemic corticosteroid treatment | 1 inotrope and no systemic corticosteroid treatment | ≥2 inotropes or 1 inotrope and systemic corticosteroid treatment | ≥2 inotropes and systemic corticosteroid treatment |
| **Hematologic score** | 0 | 1 | 2 | 3 | NA |
| Criteria^c^ | Platelet count  ≥150×10^3^ | Platelet count  100-149×10^3^ | Platelet count  <100×10^3^ | Platelet count  <50×10^3^ |  |
| **Gestational age score** | 0 | 2 | 4 | 6 | NA |
| Criteria | 37-42 | 32-36 | 29-31 | 23-28 |  |

**Abbreviations:** nSOFA: feonatal sequential organ failure assessment; FiO_2_: fraction of inspiratory oxygen; SpO_2_: peripheral oximetric saturation.

*SI conversion factor: To convert platelet count to ×10^9^/L, multiply by 1.

^a^ Score range, 0 (best) to 15 (worst).

^b^ Medications considered as inotropic or vasoactive included dopamine, dobutamine, epinephrine, norepinephrine, vasopressin, and phenylephrine.

^c^ Most recent platelet count available to the clinician.

**Table S3 Missing Number (%) for Included Variables in Dataset**

| **Characteristic** | **Missing (%)** |  | **Characteristic** | **Missing (%)** |
| --- | --- | --- | --- | --- |
| **Demographic** |  |  | **Laboratory Tests** |  |
| Ethnicity, n (%) | 0(0.0) |  | INR | 1194(93.2) |
| Sex, male, n (%) | 0(0.0) |  | PT, seconds | 1194(93.2) |
| Gestational age, n (%) | 71(5.5) |  | PH | 383(29.9) |
| Height, cm | 57(4.4) |  | PaO_2_, mmHg | 383(29.9) |
| Weight, kg | 30(2.3) |  | PaCO_2_, mmHg | 384(30.0) |
| Head circumference, cm | 48(3.7) |  | **Comorbidities** |  |
| **Vital Signs** |  |  | AKI (48h), n (%) | 0(0.0) |
| PaO_2_/FiO_2_ | 415(32.4) |  | HDN, n (%) | 0(0.0) |
| SpO2/FiO2 | 415(32.4) |  | CHD, n (%) | 0(0.0) |
| HR, bmp | 47(3.7) |  | Acidosis, n (%) | 0(0.0) |
| Temperature, ℃ | 1247(97.3) |  | Anemia, n (%) | 0(0.0) |
| Respirate, /minute | 46(3.6) |  | Bleed, n (%) | 0(0.0) |
| Urine output, ml/d | 105(8.2) |  | Pneumonia, n (%) | 0(0.0) |
| **Laboratory Tests** |  |  | Jaundice, n (%) | 0(0.0) |
| RBC, m/uL | 26(2.0) |  | Septicemia, n (%) | 0(0.0) |
| Hemoglobin, g/dL | 23(1.8) |  | **Medications** |  |
| Reticulocyte, % | 768(60.0) |  | Ventilation time, hours | 90(7.0) |
| RDW, % | 28(2.2) |  | Mechanical ventilation, n (%) | 0(0.0) |
| Hematocrit, % | 33(2.6) |  | Methasone, n (%) | 0(0.0) |
| WBC, K/uL | 17(1.3) |  | Microbiotic, n (%) | 0(0.0) |
| Lymphocyte, % | 18(1.4) |  | Furosemide, n (%) | 0(0.0) |
| Neutrophil, % | 19(1.5) |  | Dopamine, n (%) | 0(0.0) |
| Platelet, K/uL | 22(1.7) |  | Dobutamine, n (%) | 0(0.0) |
| GLU, mg/dL | 1210(94.5) |  | Epinephrine, n (%) | 0(0.0) |
| Creatinine, mg/dL | 812(63.4) |  | pulmonary surfactant, n (%) | 0(0.0) |
| TG, mg/dL | 709(55.3) |  | **Events** |  |
| Albumin, g/dL | 1143(89.2) |  | Death, n (%) | 0(0.0) |
| ALP, IU/L | 764(59.6) |  | **Score** |  |
| Bilirubin, mg/dL | 48(3.7) |  | nSOFA | 0(0.0) |
| BUN, mg/dL | 805(62.8) |  | Respiratory score | 0(0.0) |
| Serum potassium, mEq/L | 80(6.2) |  | Cardiovascular score | 0(0.0) |
| Serum sodium, mEq/L | 78(6.1) |  | Hematological score | 0(0.0) |

**Abbreviations:** FiO_2_: fraction of inspiratory oxygen; PaO_2_: partial pressure of arterial oxygen; SpO_2_: peripheral oxygen saturation; HR, heart rate; bmp, beats per minute; RBC, red blood cell; RDW, red cell distribution width; WBC, white blood cell; GLU, glucose; TG, triglyceride; ALP, alkaline phosphatase; BUN, blood urea nitrogen; INR, International Normalized Ratio; PT, prothrombin time; PH, pondus hydrogenii; PaCO_2_, partial pressure of carbon dioxide; AKI, acute renal injury; HDN, hemolytic disease of the newborn; CHD, congenital heart disease; nSOFA, neonatal sequential organ failure assessment.

**Table S4 Demographic Data and Comparisons between Survivors and Non-Survivors**

| **Characteristic** | **Original Cohort** | | | ***P-value*** |  | **Matched Cohort** | | | ***P-value*** |
| --- | --- | --- | --- | --- | --- | --- | --- | --- | --- |
|  | **Overall** | **Survivors** | **Non-survivors** |  |  | **Overall** | **Survivors** | **Non-survivors** |  |
|  | (N=1,281) | (N=1,241) | (N=40) |  |  | (N=72) | (N=36) | (N=36) |  |
| **Demographic** | | | | | | | | | |
| Ethnicity, n (%) |  |  |  | 0.421 |  |  |  |  | 1.000 |
| Asian | 48 (3.7) | 47 (3.8) | 1 (2.5) |  |  | 0 (0.0) | 0 (0.0) | 0 (0.0) |  |
| Black | 148 (11.6) | 140 (11.3) | 8 (20.0) |  |  | 14 (19.4) | 7 (19.4) | 7 (19.4) |  |
| Hispanic/Latino | 55 (4.3) | 53 (4.3) | 2 (5.0) |  |  | 2 (2.8) | 1 (2.8) | 1 (2.8) |  |
| White | 849 (66.3) | 827 (66.6) | 22 (55.0) |  |  | 42 (58.3) | 21 (58.3) | 21 (58.3) |  |
| Other | 181 (14.1) | 174 (14.0) | 7 (17.5) |  |  | 14 (19.4) | 7 (19.4) | 7 (19.4) |  |
| Sex, male, n (%) | 733 (57.2) | 711 (57.3) | 22 (55.0) | 0.900 |  | 42 (58.3) | 21 (58.3) | 21 (58.3) | 1.000 |
| Gestational age, n (%) |  |  |  | <0.001 |  |  |  |  | 1.000 |
| 23-25 weeks | 35 (2.9) | 19 (1.6) | 16 (47.1) |  |  | 26 (36.1) | 13 (36.1) | 13 (36.1) |  |
| 26-28 weeks | 101 (8.3) | 91 (7.7) | 10 (29.4) |  |  | 20 (27.8) | 10 (27.8) | 10 (27.8) |  |
| 29-31 weeks | 346 (28.6) | 343 (29.2) | 3 (8.8) |  |  | 6 (8.3) | 3 (8.3) | 3 (8.3) |  |
| 32-35 weeks | 642 (53.1) | 638 (54.3) | 4 (11.8) |  |  | 8 (11.1) | 4 (11.1) | 4 (11.1) |  |
| 36 weeks | 3 (0.2) | 3 (0.3) | 0 (0.0) |  |  | 0 (0.0) | 0 (0.0) | 0 (0.0) |  |
| 37 weeks | 69 (5.7) | 69 (5.9) | 0 (0.0) |  |  | 0 (0.0) | 0 (0.0) | 0 (0.0) |  |
| 38 weeks | 1 (0.1) | 1 (0.1) | 0 (0.0) |  |  | 0 (0.0) | 0 (0.0) | 0 (0.0) |  |
| 39 weeks | 0 (0.0) | 0 (0.0) | 0 (0.0) |  |  | 0 (0.0) | 0 (0.0) | 0 (0.0) |  |
| ≥ 40 weeks | 13 (1.1) | 12 (1.0) | 1 (2.9) |  |  | 2 (2.8) | 1 (2.8) | 1 (2.8) |  |
| Height, cm | 40.70 (6.35) | 40.90 (6.27) | 34.17 (5.60) | <0.001 |  | 35.11 (5.59) | 35.78 (5.43) | 34.43 (5.75) | 0.330 |
| Weight, kg | 1.64 (0.74) | 1.66 (0.74) | 0.94 (0.56) | <0.001 |  | 1.03 (0.66) | 1.10 (0.74) | 0.98 (0.58) | 0.470 |
| Head circumference, cm | 28.73 (3.72) | 28.87 (3.64) | 24.07 (3.12) | <0.001 |  | 25.12 (3.91) | 25.96 (4.53) | 24.32 (3.09) | 0.089 |
| **Vital Signs** | | | | | | | | | |
| PaO_2_/FiO_2_ | 242.68 (146.09) | 247.14 (146.94) | 148.21 (82.82) | <0.001 |  | 164.92 (86.74) | 191.63 (84.36) | 144.31 (83.98) | 0.032 |
| SpO_2_/FiO_2_ | 267.85 (122.71) | 271.60 (123.43) | 188.50 (69.57) | <0.001 |  | 202.53 (72.86) | 224.97 (70.86) | 185.22 (70.55) | 0.032 |
| HR, bmp | 145.72 (10.04) | 145.46 (9.66) | 153.27 (16.35) | <0.001 |  | 151.43 (15.32) | 148.97 (12.75) | 153.55 (17.12) | 0.225 |
| Respirate, /minute | 44.93 (16.21) | 45.01 (16.24) | 41.94 (14.95) | 0.276 |  | 41.86 (15.51) | 40.91 (16.04) | 42.84 (15.14) | 0.625 |
| Urine output, ml/d | 87.41 (45.54) | 88.75 (45.20) | 38.10 (27.29) | <0.001 |  | 52.56 (40.11) | 66.48 (45.03) | 38.14 (28.45) | 0.007 |
| **Laboratory Tests** | | | | | | | | | |
| RBC, m/uL | 4.23 (0.67) | 4.24 (0.67) | 3.79 (0.71) | <0.001 |  | 3.85 (0.62) | 3.90 (0.50) | 3.79 (0.73) | 0.473 |
| Hemoglobin, g/dL | 15.73 (2.23) | 15.76 (2.21) | 14.64 (2.76) | 0.002 |  | 14.72 (2.50) | 14.84 (2.14) | 14.59 (2.83) | 0.675 |
| RDW, % | 17.09 (1.36) | 17.11 (1.35) | 16.64 (1.53) | 0.033 |  | 16.66 (1.27) | 16.64 (0.90) | 16.69 (1.56) | 0.887 |
| Hematocrit, % | 46.49 (7.07) | 46.70 (6.83) | 39.97 (10.67) | <0.001 |  | 41.55 (9.26) | 43.60 (6.65) | 39.55 (10.97) | 0.065 |
| WBC, K/uL | 10.82 (6.21) | 10.86 (6.22) | 9.56 (5.90) | 0.197 |  | 9.95 (7.47) | 10.44 (8.72) | 9.48 (6.11) | 0.591 |
| Lymphocyte, % | 58.02 (17.86) | 58.00 (17.79) | 58.59 (20.00) | 0.838 |  | 59.03 (17.11) | 57.54 (15.60) | 60.47 (18.57) | 0.474 |
| Neutrophil, % | 29.60 (16.32) | 29.65 (16.33) | 28.17 (15.95) | 0.577 |  | 27.90 (14.79) | 28.83 (14.42) | 26.99 (15.29) | 0.604 |
| Platelet, K/uL | 256.37 (83.37) | 257.47 (83.10) | 221.87 (85.62) | 0.009 |  | 246.23 (105.15) | 272.31 (115.87) | 220.86 (87.92) | 0.038 |
| Bilirubin, mg/dL | 5.12 (1.92) | 5.19 (1.89) | 2.47 (0.96) | <0.001 |  | 2.95 (1.27) | 3.34 (1.42) | 2.51 (0.90) | 0.008 |
| Serum potassium, mEq/L | 4.97 (1.07) | 4.97 (1.07) | 4.92 (0.99) | 0.783 |  | 4.87 (1.14) | 4.85 (1.28) | 4.89 (1.00) | 0.900 |
| Serum sodium, mEq/L | 138.76 (4.99) | 138.69 (4.92) | 141.26 (6.57) | 0.003 |  | 139.64 (6.94) | 138.32 (6.89) | 141.03 (6.82) | 0.114 |
| **Comorbidities** | | | | | | | | | |
| AKI (48h), n (%) | 52 (4.1) | 47 (3.8) | 5 (12.5) | 0.019 |  | 6 (8.3) | 1 (2.8) | 5 (13.9) | 0.201 |
| HDN, n (%) | 14 (1.1) | 14 (1.1) | 0 (0.0) | 1.000 |  | 2 (2.8) | 2 (5.6) | 0 (0.0) | 0.473 |
| CHD, n (%) | 52 (4.1) | 52 (4.2) | 0 (0.0) | 0.360 |  | 4 (5.6) | 4 (11.1) | 0 (0.0) | 0.123 |
| Acidosis, n (%) | 84 (6.6) | 80 (6.4) | 4 (10.0) | 0.569 |  | 7 (9.7) | 3 (8.3) | 4 (11.1) | 1.000 |
| Anemia, n (%) | 294 (23.0) | 284 (22.9) | 10 (25.0) | 0.903 |  | 21 (29.2) | 11 (30.6) | 10 (27.8) | 1.000 |
| Bleed, n (%) | 33 (2.6) | 28 (2.3) | 5 (12.5) | <0.001 |  | 6 (8.3) | 1 (2.8) | 5 (13.9) | 0.201 |
| Pneumonia, n (%) | 26 (2.0) | 25 (2.0) | 1 (2.5) | 1.000 |  | 1 (1.4) | 0 (0.0) | 1 (2.8) | 1.000 |
| Jaundice, n (%) | 1105 (86.3) | 1084 (87.3) | 21 (52.5) | <0.001 |  | 48 (66.7) | 30 (83.3) | 18 (50.0) | 0.006 |
| Septicemia, n (%) | 159 (12.4) | 149 (12.0) | 10 (25.0) | 0.027 |  | 22 (30.6) | 13 (36.1) | 9 (25.0) | 0.443 |
| **Medications** | | | | | | | | | |
| Ventilation time, hours | 267.51 (458.79) | 269.48 (458.68) | 210.80 (464.34) | 0.427 |  | 476.13 (654.05) | 774.66 (696.20) | 210.76 (485.48) | <0.001 |
| Mechanical ventilation, n (%) | 971 (79.3) | 932 (78.7) | 39 (97.5) | 0.007 |  | 63 (91.3) | 28 (84.8) | 35 (97.2) | 0.163 |
| Methasone, n (%) | 61 (4.8) | 59 (4.8) | 2 (5.0) | 1.000 |  | 6 (8.3) | 4 (11.1) | 2 (5.6) | 0.670 |
| Microbiotic, n (%) | 831 (64.9) | 805 (64.9) | 26 (65.0) | 1.000 |  | 48 (66.7) | 23 (63.9) | 25 (69.4) | 0.803 |
| Furosemide, n (%) | 139 (10.9) | 135 (10.9) | 4 (10.0) | 1.000 |  | 14 (19.4) | 10 (27.8) | 4 (11.1) | 0.137 |
| Dopamine, n (%) | 170 (13.3) | 138 (11.1) | 32 (80.0) | <0.001 |  | 41 (56.9) | 12 (33.3) | 29 (80.6) | <0.001 |
| Dobutamine, n (%) | 9 (0.7) | 4 (0.3) | 5 (12.5) | <0.001 |  | 4 (5.6) | 0 (0.0) | 4 (11.1) | 0.123 |
| Epinephrine, n (%) | 2 (0.2) | 1 (0.1) | 1 (2.5) | 0.075 |  | 1 (1.4) | 0 (0.0) | 1 (2.8) | 1.000 |
| pulmonary surfactant, n (%) | 143 (11.2) | 135 (10.9) | 8 (20.0) | 0.122 |  | 18 (25.0) | 10 (27.8) | 8 (22.2) | 0.785 |
| **Score** | | | | | | | | | |
| nSOFA | 1.72 (2.33) | 1.58 (2.15) | 6.20 (3.07) | <0.001 |  | 4.68 (3.35) | 3.00 (2.65) | 6.36 (3.15) | <0.001 |
| Respiratory score | 1.16 (1.77) | 1.09 (1.70) | 3.45 (2.40) | <0.001 |  | 2.67 (2.35) | 1.78 (1.90) | 3.56 (2.44) | 0.001 |
| Cardiovascular score | 0.45 (1.07) | 0.38 (1.00) | 2.45 (1.20) | <0.001 |  | 1.79 (1.48) | 1.11 (1.45) | 2.47 (1.18) | <0.001 |
| Hematological score | 0.12 (0.44) | 0.11 (0.42) | 0.30 (0.76) | 0.007 |  | 0.22 (0.61) | 0.11 (0.32) | 0.33 (0.79) | 0.123 |

**Abbreviations:** nSOFA, neonatal sequential organ failure assessment; FiO_2_: fraction of inspiratory oxygen; PaO_2_: partial pressure of arterial oxygen; SpO_2_: peripheral oxygen saturation; HR, heart rate; bmp, beats per minute; RBC, red blood cell; RDW, red cell distribution width; WBC, white blood cell; AKI, acute renal injury; HDN, hemolytic disease of the newborn; CHD, congenital heart disease.

**Table S5 Stratified and Interaction Analyses**

| **Subgroup** | **Event** | **HR (95%CI)** | ***P*-value** | ***P* for**  **interaction** |
| --- | --- | --- | --- | --- |
| **Sex** | | | | 0.360 |
| Female | 18/548 | 1.51 (1.32-1.73) | <0.001 |  |
| Male | 22/733 | 1.58 (1.33-1.87) | <0.001 |  |
| **Septicemia** | | | | 0.636 |
| No | 30/1122 | 1.45 (1.30-1.62) | <0.001 |  |
| Yes | 10/159 | 3.29 (2.61-4.15) | <0.001 |  |

Adjusted for sex, ethnicity, gestational age, weight, heart rate, anemia, bleed, septicemia, and pulmonary surfactant.

**Figure S1 Flow Chart**

**
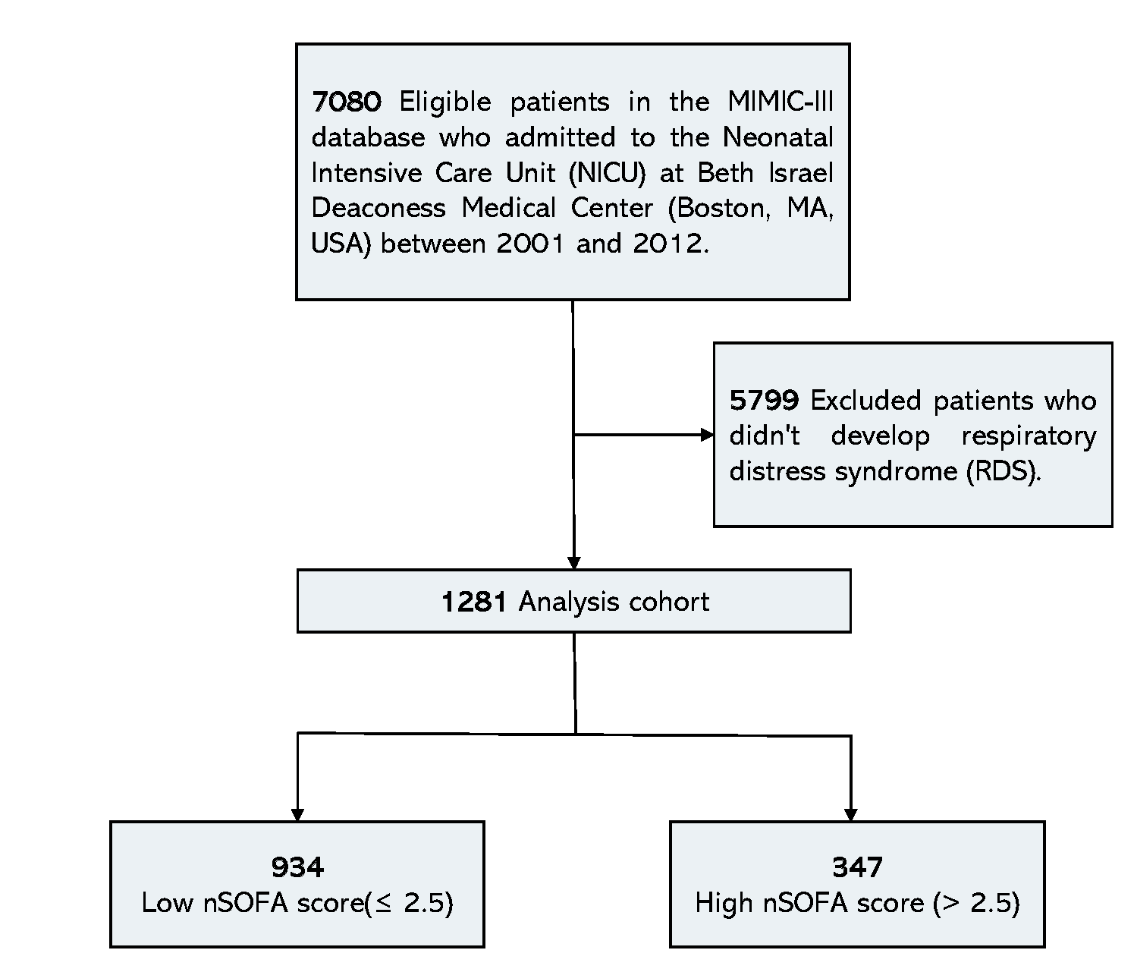
**

**Abbreviations:** MIMIC-III: Marketplace for Medical Information in Intensive Care III; NICU: neonatal intensive care unit; RDS: respiratory distress syndrome; nSOFA: neonatal sequential organ failure assessment.

**Figure S2 ROC of nSOFA Score to Predict Death in RDS Neonates**

**
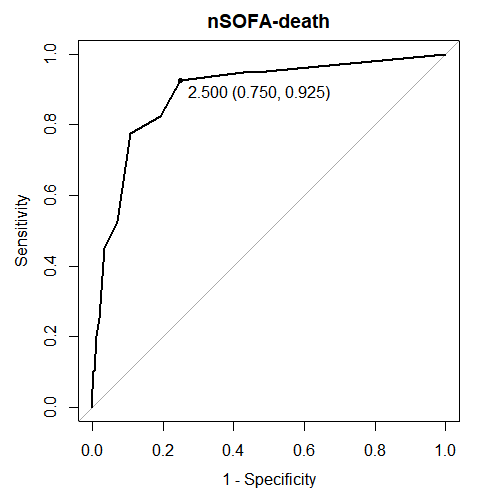
**

**Abbreviations:** ROC: receiver operating characteristic; nSOFA: neonatal sequential organ failure assessment; RDS: respiratory distress syndrome.
